# The Principles and Practice of Obstetric Medicine and Surgery

**Published:** 1845-11

**Authors:** 


### BIBLIOGRAPHICAL NOTICES.

*The Principles and Practice of Obstetric Medicine and Surgery in reference to the Process of Parturition.* Illustrated by one hundred and forty-eight figures. By FRANCIS H. RAMSBOTHAM, M.D., Fellow of the Royal College of Physicians. Consulting Physician in Obstetric Cases to, and Lecturer on Obstetrics and Forensic Medicine at the London Hospital, &c. &c. A new edition, from the enlarged and revised London edition; pp. 519; 8vo. Philadelphia: Lea & Blanchard, 1845. (From the Publishers.)

This is a reprint of the last London edition, which has been revised and much enlarged by the author. The first edition, by its extensive circulation in this country and Europe, proved its own merit. To the present edition the author has added essays on the Diseases of the Pregnant and Puerperal states, and on Abortion, with an appendix containing valuable statistical tables, founded on the Practice in the Royal Maternity Charity. The work as it now stands has been deservedly pronounced "the best authorized exponents of British Midwifery." The figures are numerous, and in design most admirably adapted to illustrate the subject, and in execution, as lithographic specimens, are scarcely to be surpassed. As a work on pure Obstetrical Science, we do not hesitate to pronounce it the most complete that it has been our pleasure to examine. With clear type, good paper, and capital execution, it forms a very handsome volume, and a most valuable addition to the library of every practitioner or student of medicine. (It may be obtained of Messrs. Brautigam & Keen, Chicago.) ED.

